# Prospective study of AI-assisted prediction of breast malignancies in physical health examinations: role of off-the-shelf AI software and comparison to radiologist performance

**DOI:** 10.3389/fonc.2024.1374278

**Published:** 2024-05-02

**Authors:** Sai Ma, Yanfang Li, Jun Yin, Qinghua Niu, Zichen An, Lianfang Du, Fan Li, Jiying Gu

**Affiliations:** ^1^ Department of Ultrasound, Shanghai General Hospital, Shanghai Jiao Tong University School of Medicine, Shanghai, China; ^2^ Department of Ultrasound, Shanghai Fourth People’s Hospital, Shanghai, China; ^3^ Department of Ultrasound, Shidong Hospital, Yangpu District, Shanghai, China

**Keywords:** ultrasound, invasive ductal carcinoma(IDC), BI-RADS ^®^, artificial intelligence (AI), incidentally diagnosed

## Abstract

**Objective:**

In physical health examinations, breast sonography is a commonly used imaging method, but it can lead to repeated exams and unnecessary biopsy due to discrepancies among radiologists and health centers. This study explores the role of off-the-shelf artificial intelligence (AI) software in assisting radiologists to classify incidentally found breast masses in two health centers.

**Methods:**

Female patients undergoing breast ultrasound examinations with incidentally discovered breast masses were categorized according to the 5^th^ edition of the Breast Imaging Reporting and Data System (BI-RADS), with categories 3 to 5 included in this study. The examinations were conducted at two municipal health centers from May 2021 to May 2023.The final pathological results from surgical resection or biopsy served as the gold standard for comparison. Ultrasonographic images were obtained in longitudinal and transverse sections, and two junior radiologists and one senior radiologist independently assessed the images without knowing the pathological findings. The BI-RADS classification was adjusted following AI assistance, and diagnostic performance was compared using receiver operating characteristic curves.

**Results:**

A total of 196 patients with 202 breast masses were included in the study, with pathological results confirming 107 benign and 95 malignant masses. The receiver operating characteristic curve showed that experienced breast radiologists had higher diagnostic performance in BI-RADS classification than junior radiologists, similar to AI classification (AUC = 0.936, 0.806, 0.896, and 0.950, *p* < 0.05). The AI software improved the accuracy, sensitivity, and negative predictive value of the adjusted BI-RADS classification for the junior radiologists’ group (*p*< 0.05), while no difference was observed in the senior radiologist group. Furthermore, AI increased the negative predictive value for BI-RADS 4a masses and the positive predictive value for 4b masses among radiologists (*p* < 0.05). AI enhances the sensitivity of invasive breast cancer detection more effectively than ductal carcinoma *in situ* and rare subtypes of breast cancer.

**Conclusions:**

The AI software enhances diagnostic efficiency for breast masses, reducing the performance gap between junior and senior radiologists, particularly for BI-RADS 4a and 4b masses. This improvement reduces unnecessary repeat examinations and biopsies, optimizing medical resource utilization and enhancing overall diagnostic effectiveness.

## Introduction

Breast cancer is now the most common malignancy in women worldwide, and its incidence continues to rise (1). Early and accurate diagnosis is crucial for improving patient outcomes. The three main imaging methods for detecting and diagnosing breast cancer are mammography, ultrasound (US), and magnetic resonance imaging (MRI).Among these methods, US is a non-radiation imaging method that has advantages for detecting breast masses, particularly in Asian women. The 5^th^ edition of the Breast Imaging Reporting and Data System (BI-RADS^®^) Lexicon published by the American College of Radiology (ACR) has standardized the description of breast masses on US and provides risk stratification for clinical decision-making, but operator dependence remains an issue. Inconsistent recognition and evaluation of breast masses by experienced and less-experienced radiologists due to varying factors, such as examination skills, experience, and equipment, can compromise the accuracy and reliability of breast cancer diagnosis, posing risks to early detection and treatment (2). According to a systematic review and meta-analysis, ultrasound screening for breast cancer has a high false-positive and recall rate, which may cause unnecessary anxiety and biopsies for women (3).

In recent years, there has been a rapid development of artificial intelligence (AI). AI can conduct a quantitative assessment by recognizing imaging information automatically and making more accurate and reproducible imaging diagnoses in breast tumors (4). AI can also save time for radiologists and compensate for the lack of experience and skill of some beginners, which can reduce subjective errors and improve diagnostic accuracy (5, 6). A recent study showed that AI applied to portable US images of breast masses could accurately identify malignancies in palpable breast masses (7). However, in clinical practice, many breast masses are incidentally found by US during physical examination. The correct classification of these incidentally discovered masses is important for making a follow-up treatment decision. In this prospective study across two medical centers, we evaluate the effectiveness of AI in diagnosing these masses and making appropriate treatment decisions.

## Materials and methods

### Patients’ enrollment

From May 2021 to May 2023, we collected data on female patients who underwent physical breast examinations at both Shanghai Fourth People’s Hospital and Shanghai General Hospital. The selection criteria were as follows: (1) Breast masses were incidentally detected during ultrasound scanning as part of a routine health examination, with no prior history of detected masses; (2) The identified breast masses were categorized as BI-RADS 3-5 upon initial examination; (3) The ultrasound images of the masses were of high quality; (4) The masses were identifiable by AI software; (5) The masses were ultimately confirmed through surgical excision or core needle biopsy. Cases that did not meet any of the inclusion criteria were excluded. The exclusion criteria comprised: (1) Patients with masses larger than 40 mm; (2) Breast masses with unclear ultrasound images; (3) Non-mass lesions in the breast; (4) Breast masses with inconclusive pathological findings.

### Acquisition of US images

The ultrasound machines used were the ACUSON Sequoia (Siemens, Germany) and Aplio500 (Canon, Japan), each equipped with a high-frequency linear array probe of 4-9 MHz and 5-14 MHz, respectively. During the scanning procedure, the mass was positioned at an appropriate depth to capture the maximum cross-section in both longitudinal and transverse views. The maximum diameter of the mass was then measured. Subsequently, the color Doppler flow imaging mode was employed, and adjustments were made to the color gain and wall filter settings to enhance the visualization of color blood flow signals within and around the mass. Still images and dynamic videos were recorded in DICOM format for further analysis.

### The interpretation of US images by radiologists with different experience

Blinded to the clinical information, pathological results and other radiological results, three breast radiologists with varying levels of experience (J.Y.G., 23 years; Y.F.L., 10 years; S.M., 3 years) independently reviewed all ultrasound images from the enrolled patients. Following the guidelines of the 5^th^ ACR BI-RADS^®^ Lexicon, the radiologists assessed the ultrasound features of breast masses and categorized them into different BI-RADS classifications (8).

The evaluation of mass characteristics was primarily based on factors such as lesion shape, orientation, margin, echo pattern, posterior features, calcifications, associated ductal changes, and color signal information. The final assessments according to the BI-RADS were as follows: 1, negative; 2, benign; 3, probably benign; 4a, low suspicion; 4b, moderate suspicion; 4c, high suspicion; or 5, highly suggestive of malignancy.

### The interpretation of US images and adjustment of BI-RADS classification by artificial intelligence-assisted diagnosis system

The AI-SONIC Breast intelligent assisted diagnosis system utilized in this study was developed by Zhejiang Deshan Yunxing Company, leveraging big data and a novel deep learning framework autonomously crafted by the company. This breast AI diagnostic system is developed based on grayscale US images of breast masses, utilizing the De-Light deep learning framework and CNNs for analysis. It starts by annotating mass features and then employs EfficientNet-B4 to analyze subtle image features and create a predictive model. The system’s training on a substantial dataset of 100,000 US images of breast masses has significantly enhanced its accuracy in distinguishing between benign and malignant masses (**Supplementary Figure**). The system’s functionality encompassed automated identification of breast masses, extraction of ultrasonic gray-scale features, and provision of probability values for benign and malignant outcomes.

To ensure consistency in the ultrasound images, two experienced radiologists with over 10 years of experience (J.Y. & F.L.) received training from the AI company’s experts. They subsequently uploaded a total of five ultrasound images of the patients’ breast masses into the system, comprising two in the transverse plane, two in the longitudinal plane, and one that exhibited typical ultrasound features. When the analysis began, the system automatically identified and examined the characteristics of the nodules, providing the BI-RADS classification and probability values for benign and malignant cases. The malignant probability value (MPV) ranged from 0 to 1, with interpretations as follows: benign (0 - 0.39), suspicious (0.4 - 0.6), and malignant (0.61 - 1). The system repeated the analysis five times, selecting the highest value as the final diagnostic result.

When adjusting the BI-RADS classification in collaboration with AI, the process involves selecting the AI’s classification when it exceeds that of the radiologists. If the AI’s diagnostic classification matches that of the radiologists, no adjustments are necessary. However, if the AI’s classification is lower than that of the radiologists, the radiologists will review the images again and provide a second assessment. A threshold of BI-RADS 4b and above was set as the point for predicting breast malignancies.

### Statistical analysis

IBM SPSS 26.0 software (IBM, USA) was used for statistical analysis. The diagnostic sensitivity, specificity, and accuracy of breast masses by different seniority radiologists and AI software were respectively analyzed and performed. ROC curves were performed by R software 4.2.1 (R Foundation for Statistical Computing, Vienna, Austria). Chi-square test was used for categorical data, and t-test was used for continuous data. The *p*value < 0.05 was considered as statistically significant difference.

## Results

### The clinical and pathological information of enrolled patients

**Figure 1:**
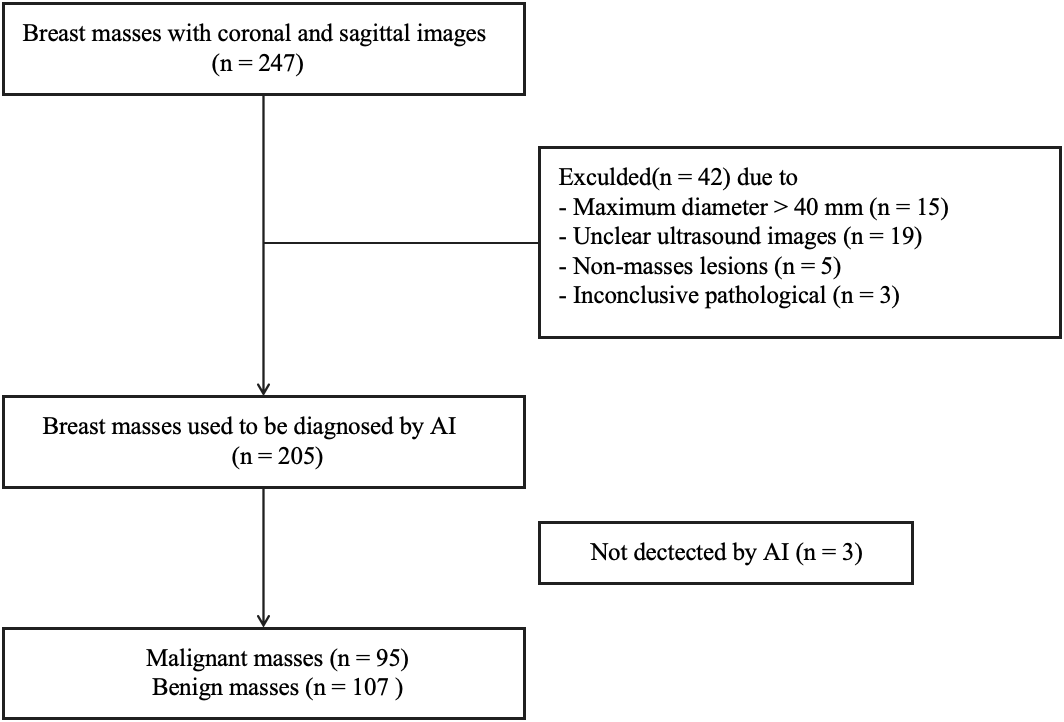
Patients’ inclusion flowchart.

According to the inclusion and exclusion criteria, a total of 196 female patients with 202 breast masses were finally enrolled in this study (**Figure 1**). The age of patients with breast malignancies was significantly higher than patients with benign masses (59.69 ± 13.18 years vs. 44.03 ± 14.09 years,*p* = 0.000). The longitudinal and transverse diameters of malignant masses were larger than those of benign masses (13.02 ± 5.46 nm vs. 8.41 ± 4.23 nm, 19.59 ± 9.10 nm vs. 14.55 ± 7.63 nm, *p* = 0.000).

The pathological result confirmed 95 malignant breast masses and 107 benign masses. The 95 malignancies included 78 invasive ductal carcinoma (IDC), 10 intraductal carcinoma *in situ* (DCIS), 2 mucinous carcinoma, 1 solid papillary carcinoma, 1 encapsulated papillary carcinoma, 1 invasive micropapillary carcinoma and 2 malignant phyllodes tumors. The 107 benign masses included 68 fibroadenomas, 21 adenosis, 3 intraductal papillomas, 2 benign phyllodes tumors, 2 granulomatous mastitis, 1 tubular adenoma, 2 atypical ductal hyperplasia, 3 sclerosing adenosis, 3 cystic lesions and 2 fibrolipomas. Out of the 107 benign masses studied, 69 underwent biopsy, while 38 underwent resection. Among the malignant cases, 38 patients underwent biopsy before surgery, while the remaining cases were directly resected with an intraoperative frozen section examination to confirm the diagnosis.

### The diagnostic results and BI-RADS classification of breast masses in AI-assisted radiologists

**Figure 2:**
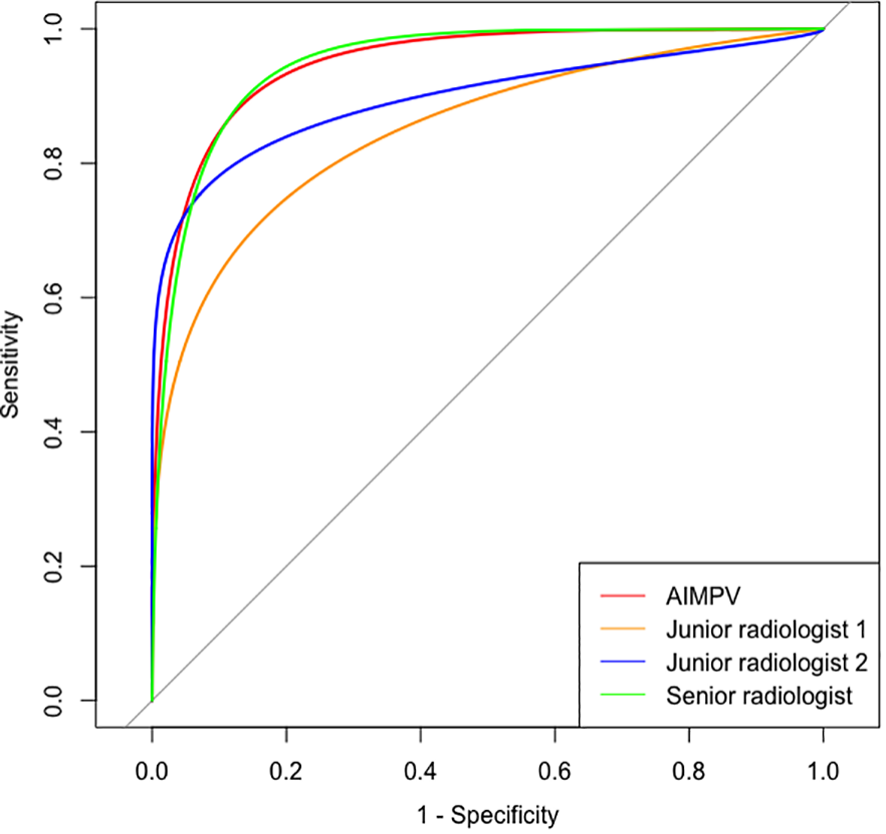
ROC curves of AI and radiologists with different experience.

The statistical analysis showed no significant difference in diagnostic sensitivity, specificity, and accuracy between the AI MPV value and AI BI-RADS classification, indicating high performance (**Table 1**). The average MPV for malignant masses was significantly higher than that for benign masses (0.803 ± 0.118 vs. 0.369 ± 0.211, *p* = 0.000). AI BI-RADS classification had a higher sensitivity (95.79%) compared to radiologists, with senior radiologists showing the highest specificity (88.79%) (**Table 2**). In ROC curve analysis, AI had the highest diagnostic efficiency with an AUC of 0.950 (*p* = 0.000), followed by senior radiologists (0.936), and junior radiologist 1 (3 years’ experience) had the lowest AUC of 0.806 (**Figure 2**).

**Table 1:** The comparison of diagnostic performance using two analyzing methods of breast AI software.

	Sensitivity	Specificity	Accuracy
AI Classification	95.79%	78.51%	86.63%
AI MPV	95.79%	79.44%	87.13%
*X* ^2^	0.000	0.028	0.022
*p*	1.000	0.867	0.883

**Table 2:** The comparison of diagnostic efficiency of AI and radiologists.

Groups	Sensitivity	Specificity	Accuracy	PPV	NPV
Junior radiologist 1	78.95%	73.83%	76.24%	72.82%	79.80%
Junior radiologist 2	85.26%	76.64%	80.69%	76.42%	85.42%
Senior radiologist	86.32%	88.79%	87.62%	87.23%	87.96%
AI	95.79%	78.51%	86.63%	79.82%	95.45%
*X* ^2^	11.845	8.354	12.121	6.663	10.373
*p1*	0.008*	0.039*	0.007*	0.083	0.016*
Junior radiologist 1 + AI	97.90%	64.49%	80.20%	70.99%	97.18%
*p2*	0.000*	0.182	0.398	0.871	0.002*
Junior radiologist 2 + AI	97.90%	74.77%	85.64%	77.50%	97.56%
*p3*	0.004*	0.873	0.231	0.972	0.010*
Senior radiologist + AI	97.90%	77.57%	87.13%	79.49%	97.65%
*p4*	0.007*	0.044*	1	0.193	0.026*

*The asterisk designates the statistically significant features if p value is less than 0.05 and the numbers are in bold.

*p1*:Comparison between the three radiologists and AI.

*p2*:Comparison of diagnostic efficiency before and after the combination of AI among Junior radiologist 1.

*p3*:Comparison of diagnostic efficiency before and after the combination of AI among Junior radiologist 2.

*p4*:Comparison of diagnostic effectiveness before and after the Senior radiologist combined AI.

The integration of AI software enhanced the diagnostic sensitivity and negative predictive value of all three radiologists (all *p* < 0.05). The combination with AI led to improved diagnostic accuracy for both junior radiologists 1 (3 years’ experience) (76.24% vs. 80.20%, *p* = 0.398) and junior radiologists 2 (10 years’ experience) (80.69% vs. 85.64%, *p* = 0.231). The accuracy of the latter group approached that of AI and senior radiologist (86.63% and 87.62, respectively) as shown in **Table 2**.

### The diagnostic performance of AI-assisted radiologists in different subcategory and pathological subtypes of breast masses

The results showed that radiologists are more likely to label non-cancerous cases as BI-RADS 4a and 4b than the AI system. When AI was used to help with the assessment, the accuracy in identifying non-cancerous cases in BI-RADS 4a improved for all three radiologists. However, for BI-RADS 4b masses, the accuracy in correctly identifying cancerous cases decreased for one junior and one senior radiologist, as shown in **Table 3**.

**Table 3:** The diagnostic performances of BI-RADS 4 masses in AI-assisted radiologists.

Groups	NPV for BI-RADS 4a masses	PPV for BI-RADS 4b masses	PPV for BI-RADS 4c masses
Junior radiologist 1	78.40%	60.50%	96.96%
Junior radiologist 1 + AI	96.77%	33.33%	89.23%
*p*	0.003*	0.006*	0.351
Junior radiologist 2	79.41%	51.02%	96.77%
Junior radiologist 2 + AI	96.00%	31.57%	86.27%
*p*	0.019*	0.109	0.242
Senior radiologist	70.73%	77.77%	100%
Senior radiologist + AI	93.55%	47.05%	89.65%
*p*	0.033*	0.006*	0.213

*The asterisk designates the statistically significant features if p value is less than 0.05 and the numbers are in bold.

NPV represents negative predictive value, while PPV represents positive predictive value, as the threshold of BI-RADS 4b and above was set as the point for predicting breast malignancies.

Looking at the rates of cancer found, the junior and senior radiologists initially reported cancer rates of 21.59%, 20.59%, and 29.27% for BI-RADS 4a cases. In comparison, the AI system found a lower cancer rate of 18.75% for the same category. With the help of AI, the cancer rates for BI-RADS 4a cases dropped significantly (to 3.22%, 4%, and 6.45%, respectively), as detailed in the **Supplementary Table**. For BI-RADS 4b cases, the cancer rates initially identified by the junior and senior radiologists were 60.56% and 77.78%, which decreased to 33.33% and 47.06% with AI assistance, respectively.

The study also explores how AI assistance affects the diagnosis of different types of breast cancer, including IDC, DCIS, and rare breast cancers. The results, presented in **Table 4**, show that AI improved the ability to detect IDC for all radiologists but did not significantly change the detection rates for DCIS and rare cancers.

**Table 4:** The diagnostic sensitivity of various breast cancer pathological subtypes in AI-assisted radiologists.

Groups	IDC	DCIS	Rare carcinomas
Junior radiologist 1	83.33%	70.00%	42.85%
Junior radiologist 1 + AI	100%	80%	100%
*p*	0.000*	1	0.069
Junior radiologist 2	89.74%	70.00%	57.14%
Junior radiologist 2 + AI	100%	80%	100%
*p*	0.006*	1	0.192
Senior radiologist	91.02%	70.00%	57.14%
Senior radiologist + AI	100%	80%	100%
*p*	0.013*	1	0.192

*The asterisk designates the statistically significant features if p value is less than 0.05 and the numbers are in bold.

## Discussion

**Figure 3:**
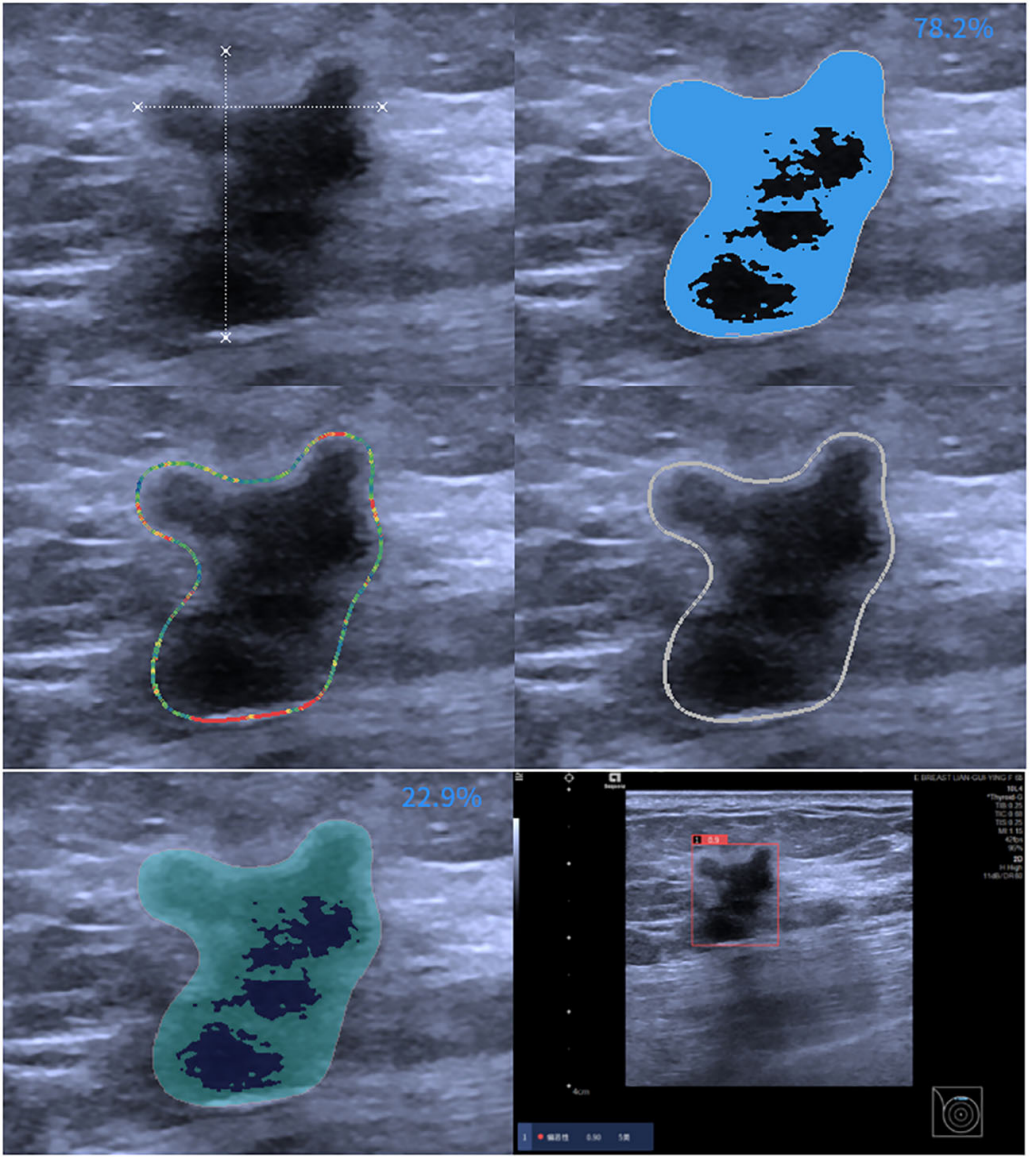
AI assessment of invasive ductal carcinoma in the right breast of a 59-year-old female patient. The AI analyzed image characteristics, including morphology, echogenicity, edge features, echo points, and solid components, resulting in a diagnosis of BI-RADS 5 with a malignancy probability of 0.9.

Ultrasound (US) is vital for breast cancer screening, especially in dense breasts (9). However, US interpretation can vary among radiologists, affecting diagnostic consistency (10). AI technology, utilizing big data and deep learning algorithms, addresses this issue by providing objectivity, stability, and high repeatability (11–13). Previous studies have shown improved diagnostic outcomes in breast masses using AI, with an average sensitivity of 84% and specificity of 85.67% (14). Our study utilized an off-the-shelf AI software system, offering high performance and safety by analyzing images in detail and automatically providing BI-RADS and MPV scores for rapid decision support in diagnosing breast masses across all hospital levels (**Figure 3**).

**Figure 4:**
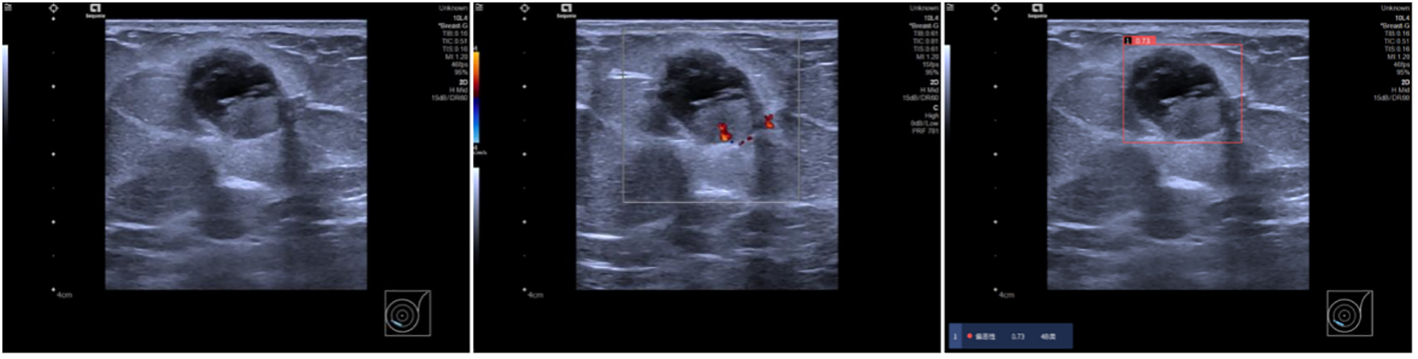
Ultrasound findings in a 69-year-old female patient showing solid cystic masses at 7-8 o’clock positions in the left breast with clear boundaries, intact envelopes, and punctate blood flow signals. Radiologists categorized the lesions as BI-RADS 4a, while AI diagnosed them as BI-RADS 4b with a malignant probability value of 0.73. Pathology confirmed left breast sternal papillary carcinoma and partial invasive carcinoma.

This study investigated the effectiveness of AI-assisted radiologists of varying experience levels in predicting malignancy in incidentally discovered breast masses. The findings suggest that AI assistance enhances the ability of junior radiologists to predict malignancy, bringing their performance closer to that of senior radiologists. AI’s utilization of machine learning algorithms helps overcome challenges in interpreting ultrasound images, providing more accurate diagnostic information (15, 16). Moreover, radiologists often classify many benign cases as BI-RADS 4a and 4b, which may due to the subjective interpretation of subtle imaging findings, variability in individual radiologists’ experience and expertise, and the inherent complexity of distinguishing between benign and malignant lesions contribute to the classification of benign cases as BI-RADS 4a and 4b (17), while AI’s advanced algorithms facilitate better distinguish benign masses from BI-RADS 4a lesions, as depicted in **Figure 4**. The malignancy rate of BI-RADS 4a after the combination of radiologists and AI is closer to the malignancy probability range of ACR BI-RADS (2%-10%) (8).The AI software may be able to assist radiologists in the diagnosis of BI-RADS 4a masses, reducing the rate of missed diagnosis of malignant breast masses.

**Figure 5:**
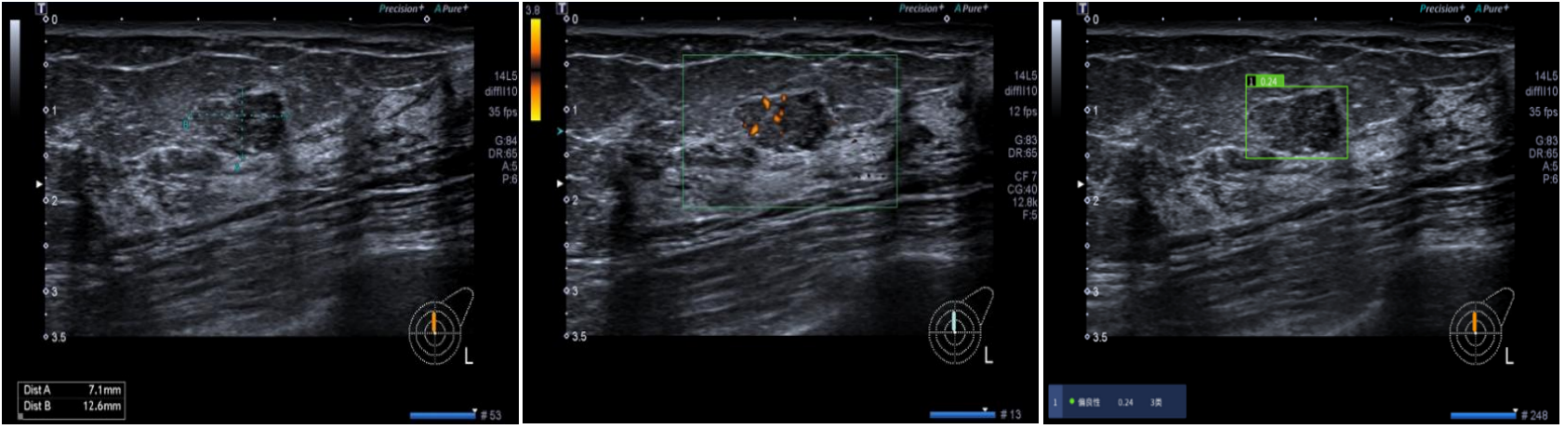
A 52-year-old female patient presented with a solid hypoechoic nodule located at 12 o’clock in the left breast, characterized by well-defined borders, intact periphery, heterogeneous echogenicity, and scattered blood flow signals. While three radiologists categorized it as 4a, the AI diagnosis indicated category 3 with a malignant probability value of 0.24. Subsequent pathology confirmed ductal carcinoma *in situ* of the breast.

This study further analyzed the efficiency of AI assistance in identifying different pathological subtypes of breast masses, demonstrating that AI primarily enhances the sensitivity of invasive breast cancer detection, with limited impact on DCIS and rare subtypes of breast cancer, as shown in **Figure 5**. IDC typically presents with more distinct imaging features, such as irregular borders, spiculated margins, microcalcifications, and architectural distortion on ultrasounds, compared to the less pronounced features seen in DCIS. The current focus of many AI software applications on IDC has created a gap in tools tailored for detecting and characterizing DCIS lesions, highlighting the need for AI algorithms optimized for DCIS data to enhance breast cancer diagnosis and treatment efficacy (18, 19). The challenge of accurately identifying DCIS when it presents as a mass, with imaging features resembling benign lesions, underscores the importance of developing AI tools specifically trained to differentiate DCIS masses from benign lesions for improved diagnostic accuracy in challenging cases (20). Additionally, the study emphasizes the importance of image quality and training data distribution in optimizing AI performance, particularly in scenarios with limited atypical mass images that may affect AI judgment accuracy.

This study has several limitations. Firstly, the sample size was relatively small, encompassing only two institutions. Future research should include a larger, multi-center patient cohort to validate the effectiveness of the AI system. Secondly, it is crucial to incorporate more DCIS and high-risk lesion images into the AI system are training dataset. This expanded training will improve the system’s ability to identify precancerous and early cancerous lesions, thereby enhancing the sensitivity of breast cancer detection. Thirdly, the study focused solely on mass lesions, overlooking the growing significance of non-mass lesions. Given that many DCIS or associated high-risk lesions may present as non-mass lesions, there is a need to establish clear diagnostic criteria for non-mass lesions on ultrasound, which should be a priority for future research (21). Fourthly, the AI breast diagnostic system utilizing grayscale US images lacks color Doppler data. The inclusion of color Doppler has the potential to enhance the diagnostic outcomes of radiologists. Nonetheless, AI, grounded in grayscale big data learning, might outperform radiologists who rely on morphological features from grayscale US images and color Doppler vessel information. The integration of dual or multi-parameter US images into the AI algorithm is poised to significantly improve diagnostic accuracy.

In conclusion, the AI intelligent auxiliary diagnosis system demonstrates high diagnostic efficiency for breast masses, enhancing the diagnostic performance of junior radiologists to approach that of senior radiologists, particularly for BI-RADS 4a and 4b masses in health examination settings. This improvement aids in avoiding unnecessary repeat examinations and reducing unwarranted biopsies, thereby optimizing medical resource utilization, and enhancing overall diagnostic efficacy. These findings suggest promising clinical applications for the AI system in the field of breast mass diagnosis.

## Data availability statement

The raw data supporting the conclusions of this article will be made available by the authors, without undue reservation.

## Author contributions

SM: Data curation, Investigation, Methodology, Visualization, Writing – original draft. YL: Investigation, Methodology, Software, Visualization, Writing – original draft. JY: Data curation, Investigation, Writing – original draft. QN: Data curation, Investigation, Writing – original draft. ZA: Data curation, Investigation, Writing – original draft. LD: Conceptualization, Supervision, Writing – original draft. FL: Conceptualization, Investigation, Resources, Supervision, Writing – review & editing. JG: Conceptualization, Data curation, Investigation, Resources, Supervision, Writing – review & editing.

## Supplementary material

Supplementary Figure The illustration of the algorithm of AI-SONIC Breast intelligent assisted diagnosis system.

## References

[1] Sung H Ferlay J Siegel RL Laversanne M Soerjomataram I Jemal A. Global cancer statistics 2020: GLOBOCAN estimates of incidence and mortality worldwide for 36 cancers in 185 countries. CA Cancer J Clin. (2021) 71:209–49. doi: 10.3322/caac.21660 33538338

[2] Berg WA Bandos AI Mendelson EB Lehrer D Jong RA Pisano ED. Ultrasound as the primary screening test for breast cancer: Analysis from ACRIN 6666. J Natl Cancer Inst. (2015) 108:djv367. doi: 10.1093/jnci/djv367 26712110 PMC5943835

[3] Yang L Wang S Zhang L Sheng C Song F Wang P. Performance of ultrasonography screening for breast cancer: A systematic review and meta-analysis. BMC Cancer. (2020) 20:499. doi: 10.1186/s12885-020-06992-1 32487106 PMC7268243

[4] Uzun Ozsahin D Ikechukwu Emegano D Uzun B Ozsahin I. The systematic review of artificial intelligence applications in breast cancer diagnosis. Diagnostics. (2022) 13:45. doi: 10.3390/diagnostics13010045 36611337 PMC9818874

[5] Lei Y Zhu H Zhang J Shan H. Meta ordinal regression forest for medical image classification with ordinal labels. IEEE/CAA J Automatica Sin. (2022) 9:1233–47. doi: 10.1109/jas.2022.105668

[6] Shen YT Chen L Yue WW Xu HX. Artificial intelligence in ultrasound. Eur J Radiol. (2021) 139:109717. doi: 10.1016/j.ejrad.2021.109717 33962110

[7] Berg WA López Aldrete AL Jairaj A Ledesma Parea JC García CY McClennan RC. Toward AI-supported US triage of women with palpable breast lumps ina low-resource setting. Radiology. (2023) 307:e223351. doi: 10.1148/radiol.223351 37129492 PMC10323289

[8] Mendelson EB Böhm-Vélez M Berg WA. ACR BI-RADS Atlas: Breast imaging reporting and data system. Reston, VA: American College of Radiology (2013).

[9] Thigpen D Kappler A Brem R. The role of ultrasound in screening dense breasts—a review of the literature and practical solutions for implementation. Diagnostics. (2018) 8:20. doi: 10.3390/diagnostics8010020 29882928 PMC6023536

[10] Wei Q Zeng S Wang L Yan Y Wang T Xu J. The added value of a computer-aided diagnosis system in differential diagnosis of breast lesions by radiologists with different experience. J Ultras Med. (2021) 41:1355–63. doi: 10.1002/jum.15816 34432320

[11] Shah SM Khan RA Arif S Sajid U. Artificial intelligence for breast cancer analysis: Trends & directions. Comput Biol Med. (2022) . 142:105221. doi: 10.1016/j.compbiomed.2022.105221 35016100

[12] Lei Y Tian Y Shan H Zhang J Wang G Kalra MK. Shape and margin-aware lung nodule classification in low-dose CT images via soft activation mapping. Med Image Anal. (2020) 60:101628. doi: 10.1016/j.media.2019.101628 31865281

[13] Fruchtman Brot H Mango VL. Artificial intelligence in breast ultrasound: application in clinical practice. Ultrasonography. (2024) 43:3–14. doi: 10.14366/usg.23116 38109894 PMC10766882

[14] Brunetti N Calabrese M Martinoli C Tagliafico AS. Artificial Intelligence in breast ultrasound: From diagnosis to prognosis—a rapid review. Diagnostics. (2022) 13:58. doi: 10.3390/diagnostics13010058 36611350 PMC9818181

[15] Kuang M Li W SL C Lu XZ. Articles that use artificial intelligence for ultrasound: A reader’s guide. Front Oncol. (2021) 11:631813. doi: 10.3389/fonc.2021.631813 34178622 PMC8222674

[16] Zhang H Meng Z Ru J Meng Y Wang K. Application and prospects of AI-based radiomics in ultrasound diagnosis. Vis Comput Ind Biomed Art. (2023) 6:20. doi: 10.1186/s42492-023-00147-2 37828411 PMC10570254

[17] Niu S Huang J Li J Liu X Wang D Zhang R. Application of ultrasound artificial intelligence in the differential diagnosis between benign and Malignant breast lesions of BI-RADS 4A. BMC Cancer. (2020) 20:959. doi: 10.1186/s12885-020-07413-z 33008320 PMC7532640

[18] Lei S Zheng R Zhang S Chen R Wang S Sun K. Breast cancer incidence and mortality in women in China: Temporal trends and projections to 2030. Cancer Biol Med. (2021) 18:900–9. doi: 10.20892/j.issn.2095-3941.2020.0523 PMC8330522 34002584

[19] Scoggins ME Fox PS Kuerer HM Rauch GM Benveniste AP Park YM. Correlation between sonographic findings and clinicopathologic and biologic features of pure ductal carcinoma *in situ* in 691 patients. Am J Roentgenol. (2015) 204:878–88. doi: 10.2214/ajr.13.12221 25794082

[20] Qian L Lv Z Zhang K Wang K Zhu Q Zhou S. Application of deep learning to predict underestimation in ductal carcinoma *in situ* of the breast with ultrasound. Ann Transl Med. (2021) . 9:295. doi: 10.21037/atm-20-3981 33708922 PMC7944276

[21] Park KW Park S Shon I Kim MJ Han BK Ko EY. Non-mass lesions detected by breast US: stratification of cancer risk for clinical management. Eur Radiol. (2021) . 31:1693–706. doi: 10.1007/s00330-020-07168-y 32888070

