# Supplementary figures and images for: Prospective study of AI-assisted prediction of breast malignancies in physical health examinations: role of off-the-shelf AI software and comparison to radiologist performance
Source: Front Oncol. 2024 May 2;14:1374278. doi: 10.3389/fonc.2024.1374278 (PMC11096442; doi:10.3389/fonc.2024.1374278)

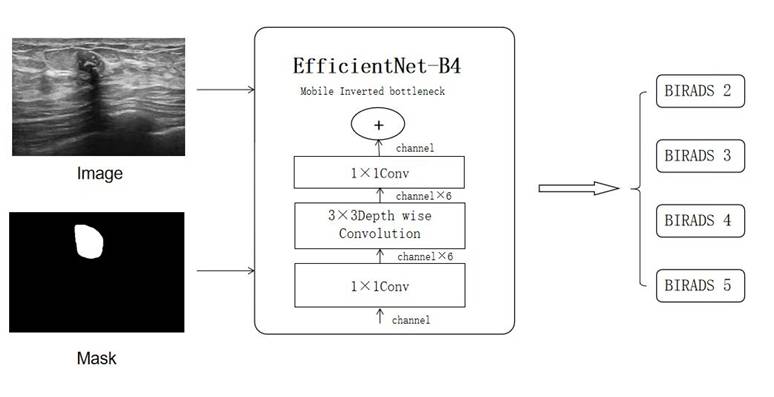

Supplement: Supplementary Figure — The illustration of the algorithm of AI-SONIC Breast intelligent assisted diagnosis system. [file Image_1.jpeg]
