# Supplementary material for: Prospective study of AI-assisted prediction of breast malignancies in physical health examinations: role of off-the-shelf AI software and comparison to radiologist performance
Source: Front Oncol. 2024 May 2;14:1374278. doi: 10.3389/fonc.2024.1374278 (PMC11096442; doi:10.3389/fonc.2024.1374278)
Supplement: Supplementary file 2 [file Table_1.docx]

Supplementary Material

**Supplementary Table The malignancy rate in BI-RADS subcategories in AI-assisted radiologists**

| Group | BI-RADS 3 | BI-RADS 4a | BI-RADS 4b | BI-RADS 4c | BI-RADS 5 |
| --- | --- | --- | --- | --- | --- |
| Junior radiologist 1 | 10.00%  (1/10) | 21.59%  (19/88) | 60.56%  (43/71) | 96.97%  (32/33) | 0.00%  (0/0) |
| Junior radiologist 1 + AI | 0.00%  (0/8) | 3.22%  (2/62) | 33.33%  (16/48) | 89.23%  (58/65) | 100%  (19/19) |
| Junior radiologist 2 | 0.00%  (0/28) | 20.59%  (14/68) | 51.02%  (25/49) | 96.77%  (30/31) | 100%  (26/26) |
| Junior radiologist 2 + AI | 0.00%  (0/26) | 4%  (2/50) | 31.58%  (12/38) | 86.27%  (44/51) | 100%  (37/37) |
| Senior radiologist | 1.49%  (1/67) | 29.27%  (12/41) | 77.78%  (42/54) | 100%  (26/26) | 100%  (14/14) |
| Senior radiologist + AI | 0.00%  (0/54) | 6.45%  (2/31) | 47.06%  (16/34) | 89.66%  (52/58) | 100%  (25/25) |
| AI | 1.38%  (1/72) | 18.75%  (3/16) | 50%  (17/34) | 90.16%  (55/61) | 100%  (19/19) |
